# miR-34c inhibits PDGF-BB-induced HAVSMCs phenotypic transformation and proliferation via PDGFR-β/SIRT1 pathway

**DOI:** 10.1007/s11033-021-06427-5

**Published:** 2021-06-10

**Authors:** Wei-feng Wan, Xin Zhang, Chang-ren Huang, Li-gang Chen, Xiao-bo Yang, Kun-yang Bao, Tang-ming Peng

**Affiliations:** 1 Department of Neurosurgery, Affiliated Hospital of Southwest Medical University, 25 Taiping Road, Luzhou, 646000 Sichuan People’s Republic of China; 2 Neurosurgery Clinical Medical Research Center of Sichuan Province, Luzhou, 646000 Sichuan People’s Republic of China; 3 Academician (Expert) Workstation of Sichuan Province, Luzhou, 646000 Sichuan People’s Republic of China; 4 Department of Neurosurgery, Luzhou People’s Hospital, Luzhou, 646010 Sichuan People’s Republic of China

**Keywords:** Vascular smooth muscle cell, miR-34c, Phenotypic transformation, Proliferation, Apoptosis

## Abstract

The purpose of this study was to explore the effect of miR-34c on PDGF-BB-induced HAVSMCs phenotypic transformation and proliferation via PDGFR-β/SIRT1 pathway, so as to find a new method for early diagnosis and treatment of cardiovascular disease. HA-VSMCs were treated with platelet-derived growth factor-BB (PDGF-BB) at 0 h, 12 h, 24 h, 48 h or 36 h to explore the optimal time for phenotypic transformation of VSMCs. And then, PDGF-BB-induced HA-VSMCs were transfected with miR-34c mimics/mimics NC and pcDNA3.1-PDGFR-β/pcDNA3.1-NC to observe cell biological behaviour. CCK8 was used to detect cell proliferation activity. Transwell chamber assay was used to detect cell invasion. Early apoptosis was analyzed by flow cytometry. The expression of α-SMA and Smemb was detected by immunofluorescence staining. The expressions of PDGFR-β, IRF9, Acetyl-NF-κB/p65, Acetyl-p53 and CyclinD1 were analyzed by Western blot analysis. The expression of miR-34a, miR-34b and miR-34c was detected by RT-PCR, and the targeting relationship between miR-34c and PDGFR-β was detected by luciferase reporting assay. The results indicated the proliferation and migration of PDGF-BB-induced HA-VSMCs significantly increased, and apoptosis significantly decreased. Besides, α-SMA decreased significantly, while Smemb increased significantly. Furthermore, expressions of PDGFR-β, IRF9, Acetyl-NF-κB/p65, Acetyl-p53 and CyclinD1 increased significantly, and SIRT1 decreased significantly. Experimental results showed that, miR-34c mimics significantly inhibited cell proliferation and migration, and promoted cell apoptosis, and miR-34c inhibitor had the opposite effects. MiR-34c mimics significantly increased α-SMA expression and decreased Smemb expression, while the opposite effects were reflected after transfection with miR-34c inhibitor. Moreover, miR-34c mimics significantly decreased the expressions of PDGFR-β, IRF9, Acetyl-NF-κB/p65, Acetyl-p53 and CyclinD1, and significantly increased the expression of SIRT1, while miR-34c inhibitor had the opposite effects. Luciferase assay confirmed that PDGFR-β was a potential target of miR-34c. Subsequently, PDGF-BB-induced HA-VSMCs were co-transfected with miR-34c mimics and pcDNA3.1-PDGFR-β. The results indicated that PDGFR-β reversed the biological function of miR-34c mimic. The results revealed the potential application value of miR-34c as a marker molecule of phenotypic transformation, providing a potential target for improving phenotypic transformation.

**Supplementary Information:**

The online version contains supplementary material available at 10.1007/s11033-021-06427-5.

## Introduction

Abnormal proliferation of vascular smooth muscle cells (VSMC) is the common pathological basis of many cardiovascular diseases. The transformation of the VSMC in the vascular median membrane from differentiation to dedifferentiation is the prerequisite for its proliferation, and this transformation is called the phenotypic transformation of VSMC, which plays an important role in the occurrence of vascular diseases such as atherosclerosis and restenosis [1]. Since smooth muscle cells (SMC) from the mesoderm during embryonic development, the SMC can differentiate into different cell groups to obtain a differentiated phenotype with adult characteristics. This phenotype is contractile and its main function is to maintain the elasticity of blood vessels. Therefore, phenotypic transformation of vascular SMC is the main cause of vascular remodeling, including decreased vascular elasticity and progressive vascular lumen stenosis. In addition, under the stimulation of internal environmental factors, such as platelet-derived growth factor-BB (PDGF-BB), the mature VSMC will undergo dedifferentiation and become a less differentiated secretory phenotype. Finally, it shows a series of biological effects such as abnormal proliferation, migration and apoptosis of SMC [2], which leads to insufficient blood perfusion, abnormal organ function and even failure of terminal organs [3]. Therefore, inhibiting the phenotypic transformation and proliferation of PDGF-BB induced SMC determines the occurrence, development and outcome of cardiovascular and cerebrovascular diseases such as pulmonary arterial hypertension and atherosclerosis.

miRNAs, as non-coding RNAs discovered in recent years, have the function of regulating gene expression and play an important role in cell growth, differentiation, migration and other processes, playing the role of key small molecule to regulate many pathophysiological phenomena [4]. Recent researches show that miRNAs play an important role in phenotypic transformation of vascular smooth muscle [5]. Various miRNAs are specifically expressed in VSMCs and thus regulate the proliferation and migration of VSMCs [6, 7]. Therefore, changing the expression of miRNAs may become a new target for the treatment of vascular remodeling diseases. As a highly conserved class of miRNAs, miR-34s play an important role in cells. In normal animal cells, the main functions of miR-34s are to promote cell senescence [8], induce cell apoptosis [9], and prevent cell migration [10]. Studies have shown that miR-34a is significantly increased during the differentiation of stem cells in smooth muscle cells, and it has been found that miR-34a plays an important role in promoting the differentiation of smooth muscle cells [11]. In addition, in terms of inhibiting phenotypic transformation of VSMC, early studies have found that miR-34c inhibited angiogenic intimal hyperplasia by regulating the PI3K/Akt cell signaling pathway [12]. However, the role of miR-34c in the phenotypic transformation and proliferation of PDGF-BB-induced HA-VSMC remains unknown.

Mir-34c has a wide range of functions, regulating the important processes of cell proliferation and differentiation, and is closely related to the occurrence of a variety of diseases [13, 14]. Based on the above, it can be speculated that miR-34c is an important regulator to maintain homeostasis in the body. However, currently, there are few studies on the role of miR-34c in vascular remodeling diseases such as hypertension. Previous studies have shown that platelet-derived growth factor receptor β (PDGFR-β) is expressed in VSMC of brain and is associated with spasm the pathogenesis of cerebral vasospasm after subarachnoid hemorrhage [15]. In the previous work, we found for the first time that PDGFR-β could promote phenotypic transformation of VSMCs, and the PDGFR/IRF9/SIRT1 axis was an important signaling pathway mediating VSMC phenotypic transformation [16]. Bioinformatics analysis showed that miR-34c had a target binding site with PDGFR-β. In addition, in our preliminary experiment, we found that PDGF-BB showed the best effect on reducing miR-34c rather than miR-34a/b, which was consistent with our original intention. Therefore, in view of the close correlation between phenotypic transformation of VSMC and the occurrence of various macrovascular diseases [17], we investigated whether miR-34c inhibited PDGF-BB-induced phenotypic transformation and proliferation in HA-VSMCs by regulating the PDGFR-β/SIRT1 pathway. By assessing the expression levels of a series of proteins, including α-smooth muscle actin (α-SMA), embryonic smooth muscle myosin heavy chain (Smemb) and cyclin D1, we sought to explore the role of miR-34c in PDGF-BB induced phenotypic transformation and proliferation of HA-VSMCs via PDGFR-β/SIRT1 pathway, providing new ideas for the pathogenesis of vascular remodeling diseases such as hypertension.

## Materials and methods

### Cell culture and treatment

Human aortic vascular smooth muscle cells (HA-VSMCs, Yanyu biotechnology, Shanghai, China) co. LTD. were cultured in Dulbecco’s Modifed Eagle Medium (DMEM; Gibco, Suzhou, China) with 7% fetal bovine serum (FBS; Gibco, Suzhou, China) and antibiotics (1% penicillin/streptomycin; Solarbio, Beijing, China) at 37 °C in a humidified air containing 5% CO_2_ incubator. After not more than 4–6 passages, to determine the optimal time for phenotypic transformation of VSMCs, cells were treated with 20 ng/mL PDGF-BB at 0 h, 12 h, 24 h, 48 h or 36 h once the cells were attached. Besides, to investigate the effects of overexpression or interference of miR-34c on the phenotypic transformation and proliferation of HA-VSMCs induced by PDGF-BB, 20 ng/mL PDGF-BB (T&L Biotechnology Co., Ltd., Beijing, China) was added to the transfected cells to stimulate HA-VSMCs for 24 h after serum starvation.

### Cell transfection

Until 60% of confluence, the HA-VSMC cells were transfected with 50 nmol/L of miR-34c mimics, mimics NC, miR-34c inhibitor or inhibitor NC according to the instructions of Lipofectamine 2000 (Invitrogen, ThermoFisher Scientific, CA, USA). In another experiment, the HA-VSMC cells were co-transferred with mimics NC/miR-34c mimics and pcDNA3.1-NC/pcDNA3.1-PDGFR-β according to the instructions of Lipofectamine 2000 (Invitrogen, ThermoFisher Scientific, CA, USA). The complete medium was replaced once until 6 h later. Cells transfected for 24–48 h were collected for the vitro experiments.

### CCK-8 assay

Cells were first seeded into 96-well plates and cultured overnight, and the medium was replaced after 12 h, with 5 repeating holes set in each group. Absorbance (A) at 450 nm at the appointed time points was recorded in accordance with the Cell Counting Kit-8 (CCK-8; Dojindo Laboratories, Kumamoto, Japan). Finally, the survival curve was drawn according to the cell proliferation.

### Transwell migration assay

Cell migration was evaluated in transwell tablets (Corning life sciences, USA). A total of 5 × 10^3^ cells were implanted into the upper chamber of the 24-well plate. After serum starvation, 20 ng/mL PDGF-BB was added to stimulate the smooth muscle cells 24 h later. Serum-free H-DMEM was added to the upper chamber and 10% fetal bovine serum was added to the lower chamber. After incubation at 37 °C at night, the unmigrated cells were removed with sterile cotton swabs. Migrants were fixed with frozen methanol, then dyed with crystal violet, and finally counted randomly in five regions.

### Apoptosis determination

HA-VSMC cells in logarithmic growth stage were digested by trypsin and centrifuged at 1000 r/min for 5 min. Next, the supernatant was discarded, and the cells were washed with 1 × binding buffer to precipitate for one time. Then 100 μl 1 × binding buffer were used to resuspended the cells, so that the cell density was adjusted to 1 × 10^6^ cells/mL. After that, 5 μl AnnexinV-FITC was added, followed by 5 μl PI staining, and then incubated at room temperature and away from light for 15 min.

### Immunofluorescence staining

Immunofluorescent staining was performed as described previously. Briefly, HA-VSMC cells were seeded in 24-well plates with sterile cover slides. The DMEM medium was then discarded and the cells were washed with PBS for three times. These samples were immediately fixed with 4% paraformaldehyde for 15 min, and then washed with PBS for three times. Subsequently, the membrane was then broken with 0.5% Triton x-100 for 20 min, washed with PBS, and then blocked with 2% BSA at room temperature for 30 min. HA-VSMC cells were incubated overnight at 4 °C with primary antibodies: rabit anti-α-SMA (Abcam, United Kingdom, 1:200) and rabbit anti-Smemb (Abcam, United Kingdom, 1:200), followed by incubation with fluorescein isothiocyanate-conjugated and tetramethyl rhodamine isothiocyanate-conjugated secondary antibodies (Abbkine, USA, 1:200) for 60 min at room temperature. The nucleus were stained with DAPI. No primary antibody was added to control staining. The cells were observed and photographed using an inverted fluorescence microscope (Olympus, Japan).

### Western blot

Western blot analysis was performed to detect protein expression in HA-VSMC cells. Protein extraction kit (Pierce, Thermo Fisher, Ltd.) was used to extract protein. The total protein concentration of the sample was determined by the protocol of KCTMBCA protein quantitative kit. Protein lysates were electrophoresed using SDS-PAGE and then transferred to PVDF membranes. The membranes were blocked with 5% non-fat milk powder, incubated with primary antibody: rabbit anti-PDGFR-β (ab32570, Abcam, 1:5000), rabbit anti-IRF9 (ab126940, Abcam, 1:2000), rabbit anti-Acetyl-NF-κB/p65 (#3045, CST, 1:1000), rabbit anti-Acetyl-p53 (#2570, CST, 1:1000), rabbit anti-CyclinD1 (ab226977, Abcam, 1:2000) or a mouse monoclonal β-actin antibody (ab49900, Abcam, 1/25000) overnight at 4 °C, followed by binding to horseradish peroxidase-conjugated secondary antibody (Santa Cruz Biotechnology, USA). All membranes were detected using enhanced chemiluminescence (Thermo Fisher Scientific), and the protein expression was analyzed with ImageJ (National Institutes of Health, Bethesda, MD, USA).

### Real-time PCR

Total RNA was extracted using Trizol reagent based on the manufacturer’s instructions. The equal RNA was used to reverse transcribed into cDNA according to the instructions of HiScriptQ RT SuperMix for qPCR (Vazyme, Nanjing, China). The average cycle thresholds (Ct) were employed to quantify fold-change. The 2^−△△CT^ method was reported to calculate relative gene expression levels. The primer sequences for miR-34a: ACACTCCAGCTGGGTGGCAGTGTCTTAGCTGG(Forward), TGTCGTGGAGTCGGCAA

TTC (Reverse); miR-34b: ACACTCCAGCTGGGCAATCACTAACTCCACTG(Forward); TGTCGTGGAGTCGGCAATTC(Reverse); miR-34c: ACACTCCAGCTGGAGGCAGTGTA

GTTAGCTG(Forward); TGTCGTGGAGTCGGCAATTC(Reverse).

### Luciferase assay

HASMCs cells were inoculated in 24-well plates at a density of 3 × 10^5^ cells per well. Subsequently, the cells were co-transfected with miR-34c mimic/NC-mimic and pmirGLO-PDGFR-β-WT/pmirGLO-PDGFR-β-MUT using Fluorophotinase assay kit (Promega). The 3′ UTRs of the human genes was PCR amplified using the following primers: PDGFR-β, FW 5′TCTAGAAAAGAGGGCAAATGAGATCACCTCCTGCA 3′, PDGFR-β RW 5′TCTAGATATTGAGAACCCACTCTCCCTCCTTGGA 3′, and cloned downstream of the Renilla luciferase stop codon in pMIR-Report Luc vector (Ambion). The p3′-UTR-mutant-plasmids using the following primers: PDGFR-β-MUT, FW 5′-ATGGGGGTATGGTTT. TGTCAGACCTAGCAGTGAC-3′, PDGFR-β-MUT RW 5′-GTCACTGCTAGGTCTGACAAA. ACCATACCCCCAT-3′. 24 h later, co-transfected cells were collected. Finally, luciferase activity was measured by a Dual-Luciferase reporter assay system (Promega, Madison, WI, USA).

### Statistical analysis

Data were expressed as mean ± standard deviation. SPSS 17.0 (SPSS Inc.) was used to conduct one-way analysis of variance with Tukey's multiple comparison test for the differences between groups using Tukey multiple comparison test as the post-test. P<0.05 was considered statistically significant.

## Results

### PDGF-BB promoted the proliferation and migration of HA-VSMCs and inhibited its apoptosis

**Fig. 1:**
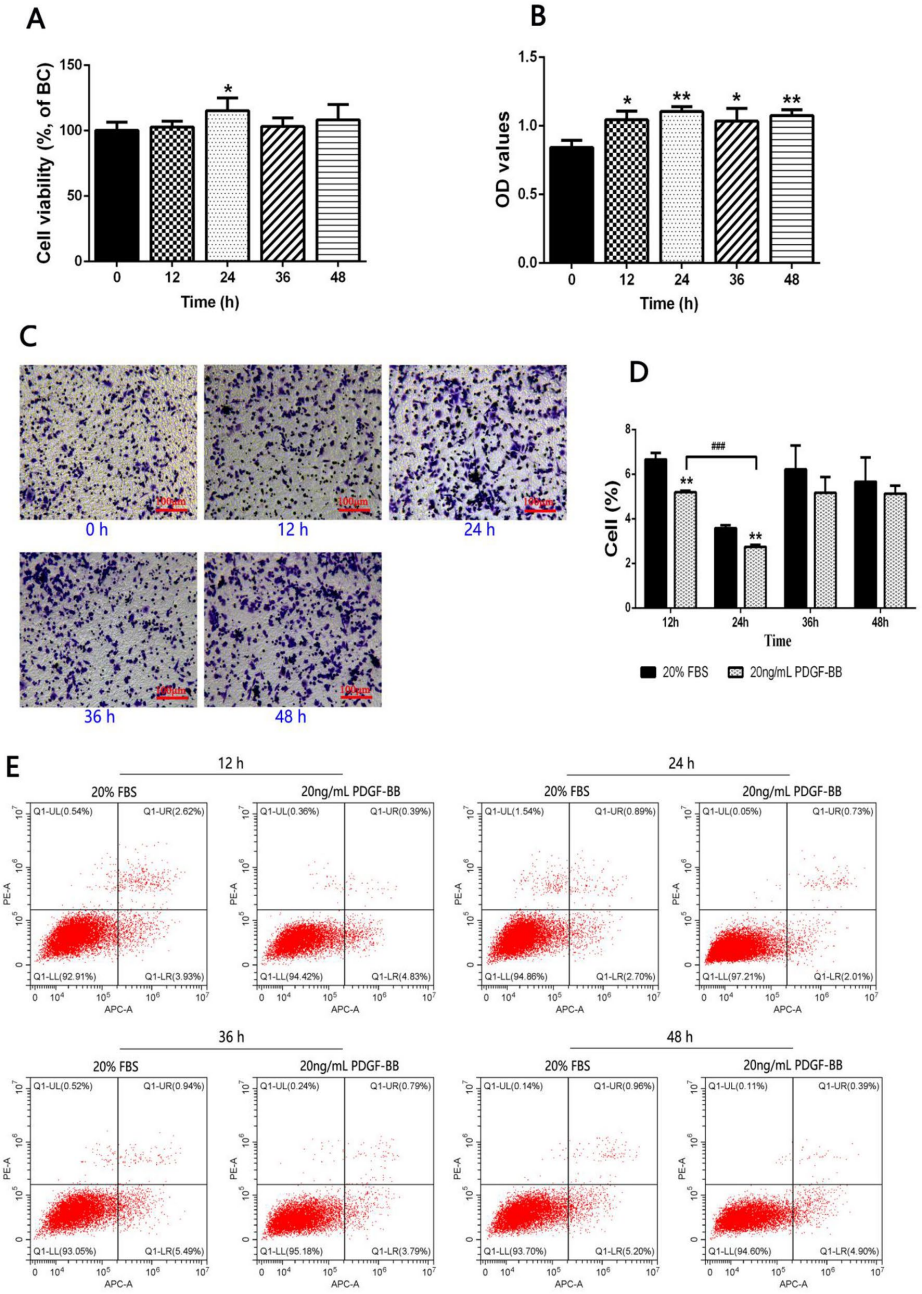
**A** HA-VSMCs viability normalized to BC (blank control, just contains 20% FBS) after treatment of 20 ng/mL PDGF-BB or 20% FBS by CCK-8 assay. *P < 0.05, vs. 0 h group. **B**, **C** HA-VSMCs were treated with PDGF-BB or 20% FBS at given time. Transwell was used to assess HA-VSMCs migration. *P < 0.05 and **P < 0.01, vs. 0 h group. **D**, **E** Apoptosis of PDGF-BB-induced HA-VSMCs were detected by flow cytometry at given time. **P < 0.01, vs. 20% FBS at the corresponding time; ^###^P < 0.001 between the comparison

In order to clarify the optimal time point for the proliferation, migration and apoptosis of HA-VSMCs cells induced by PDGF-BB, CCK8, transwell and flow cytometry experiments were carried out. The results showed that, after treatment of 20 ng/mL PDGF-BB for 24 h, the cell viability was significantly elevated relative to the treatment of 20% FBS, while with no significant difference at 0, 12, 36 and 48 h (Fig. 1A). PDGF-BB-induced HA-VSMCs migration increased significantly at 12, 24, 36 and 48 h, respectively, with an optimal time point of increase at 24 h (Fig. 1B, C). Meanwhile, compared with the cell apoptosis results after 20% FBS treatment, PDGF-BB-induced HA-VSMCs were found to have a significant decreasing trend at 12 h and 24 h, respectively, with no significant means at 36 h and 48 h (Fig. 1D, E). These data indicated that PDGF-BB promoted the proliferation and migration of HA-VSMCs and inhibited its apoptosis, and 24 h is the best time for cell survival.

### PDGF-BB induced the phenotypic transformation of HA-VSMCs through activating PDGFR-β/SIRT1 pathway

**Fig. 2:**
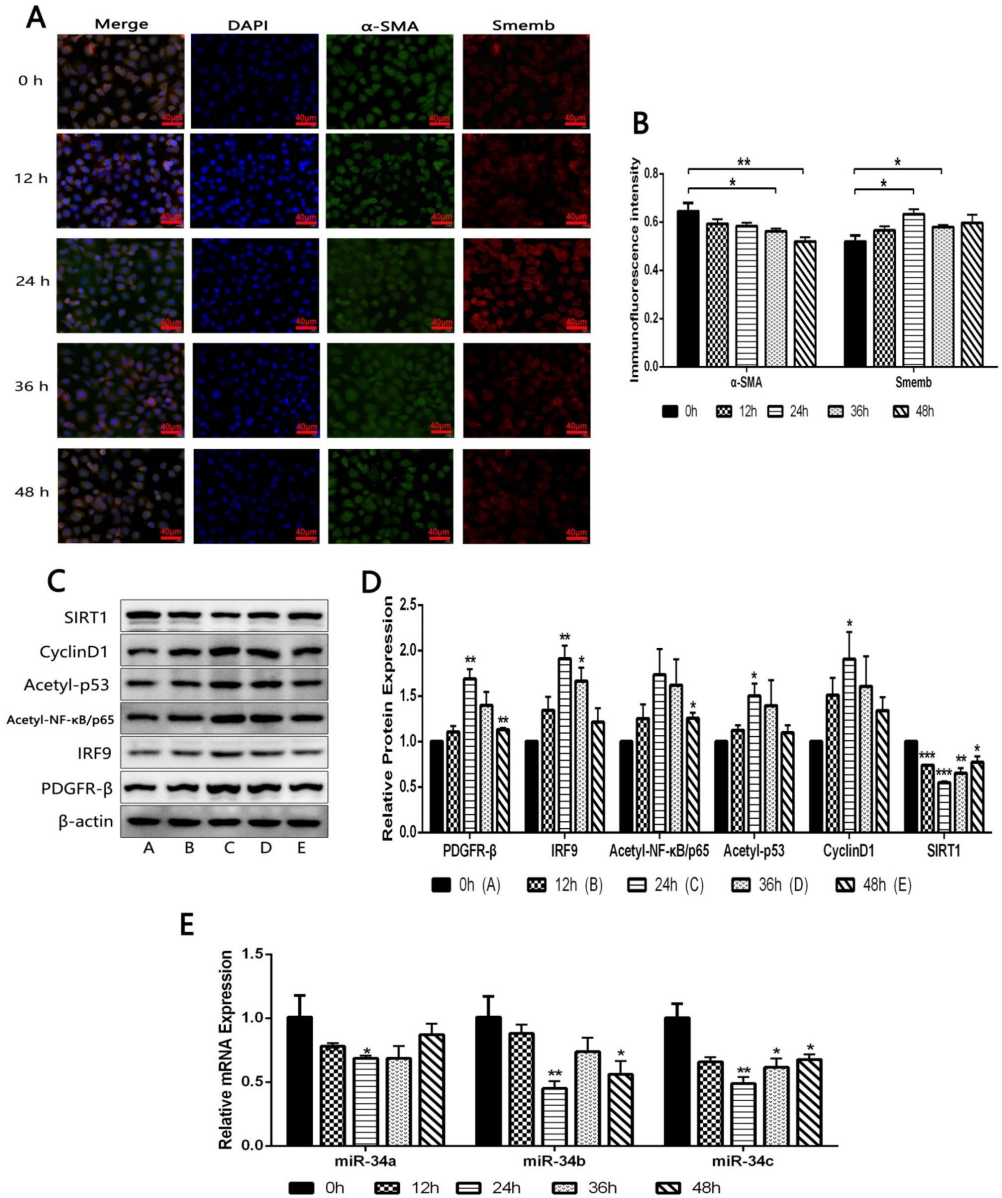
**A**, **B** Immunofluorescences of α-SMA and Smemb protein were detected in HA-VSMCs at given time after treatment of 20 ng/mL PDGF-BB by immunofluorescent staining. *P < 0.05 and **P < 0.01 between the comparison. **C**, **D** Relative protein expressions of PDGFR-β/SIRT1 pathway were detected in HA-VSMCs at given time after treatment of 20 ng/mL PDGF-BB by Western blot. *P < 0.05, **P < 0.01 and ***P < 0.001, vs. 0 h group. **E** Relative mRNA expressions of miR-34a, miR-34b and miR-34c were detected in HA-VSMCs at given time after treatment of 20 ng/mL PDGF-BB by RT-qPCR. *P < 0.05 and **P < 0.01, vs. 0 h group

Immunofluorescence was used to observe the effect of PDGF-BB treatment on phenotypic transformation-related protein expression in vascular smooth muscle cells. The results showed that, the expression level of α-SMA in PDGF-BB-induced HA-VSMCs was gradually decreased as time went on, with a significant difference at 36 h and 48 h compared to 0 h, while the expression level of Smemb was significantly increased after PDGF-BB treatment especially in 24 h and 36 h (Fig. 2A, B).

Western blot results indicated that the expression levels of PDGFR-β, IRF9, Acetyl-NF-κB/p65, Acetyl-p53 and CyclinD1 in HA-VSMCs cells exhibited an upward tendency with a peak value at 24 h after exposing to PDGF-BB, although Acetyl-NF-κB/p65 expression did not increase significantly. Conversely, compared with 0 h, the expression level of SIRT1 showed a lowest trend with a significant difference at 24 h, although there was a trend of significant decrease at any other time point (Fig. 2C, D). These data indicated that PDGF-BB induced the phenotypic transformation of HA-VSMCs through activating PDGFR-β/SIRT1 pathway.

### PDGF-BB downregulated the expression of miR-34a, miR-34b and miR-34c in HA-VSMCs

As a highly conserved class of miRNAs, miR-34s play an important role in cells. In normal animal cells, the main functions of miR-34s are to promote cell senescence. The expressions of miR-34a, miR-34b and miR-34c were first detected at different time points in PDGF-BB-induced HA-VSMCs. The results of RT-qPCR showed that compared with 0 h, the expressions of miR-34a, miR-34b and miR-34c were all down-regulated, and at 24 h, each expression level had the lowest down-regulated peak, accompanied by significant differences. In addition, miR-34c showed a significantly reduced expression at 24 h, 36 h, and 48 h, suggesting that PDGF-BB showed the best effect on reducing miR-34c rather than miR-34a/b (Fig. 2E).

### miR-34c mediated the effect of PDGF-BB on the proliferation, migration, and apoptosis of HA-VSMCs

**Fig. 3:**
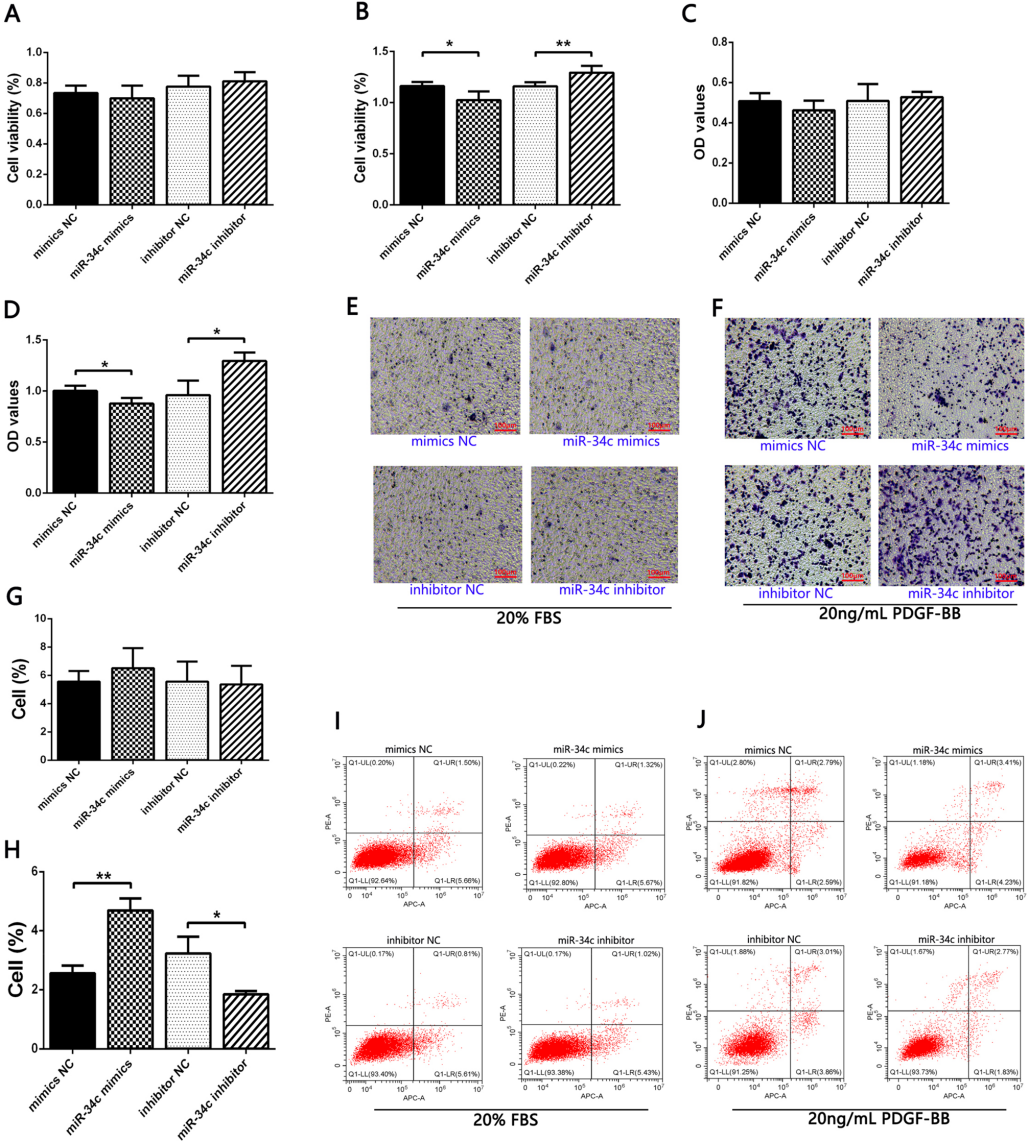
**A**, **B** CCK8 was used to detect the effects of overexpression or inhibition of miR-34c on the proliferation activity of FBS-treated (**A**) or PDGF-BB-induced (**B**) HA-VSMCs. *P < 0.05 and **P < 0.01 between the comparison. **C**–**F** Transwell migration was used to detect the effects of overexpression or inhibition of miR-34c on the migration of FBS-treated (**C** and **E**) or PDGF-BB-induced (**D** and **F**) HA-VSMCs. *P < 0.05 between the comparison. **G**–**J** Flow cytometry was used to detect the effects of overexpression or inhibition of miR-34c on the apoptosis of FBS-treated (G and I) or PDGF-BB-induced (H and J). *P < 0.05 and **P < 0.01 between the comparison

It has been found that some miRNAs can play a regulatory role in biological processes such as proliferation and migration in VSMCs. To investigate whether miR-34c regulates the biological behavior of PDGF-BB-induced HA-VSMCs, cell proliferation, migration and apoptosis were verified. Experimental results showed that, miR-34c mimics significantly inhibited cell proliferation (Fig. 3B) and migration (Fig. 3D and F), and promoted cell apoptosis (Fig. 3H and J), compared with the NC mimics group. Conversely, as expected, miR-34c inhibitor had a significant promoting effect on cell proliferation (Fig. 3B) and migration (Fig. 3D and F), as well as a significant inhibiting effect on apoptosis (Fig. 3H and J), compared with inhibitor NC group. None of these proliferation (Fig. 3A), migration (Fig. 3C and E), or apoptosis (Fig. 3G and I) outcomes were significantly different after regulation by miR-34c mimics or miR-34c inhibitor on 20% FBS cell medium without PDGF-BB stimulation.

### Inhibiting miR-34c promoted the phenotypic transformation of HA-VSMCs through activating PDGFR-β/SIRT1 Pathway

**Fig. 4:**
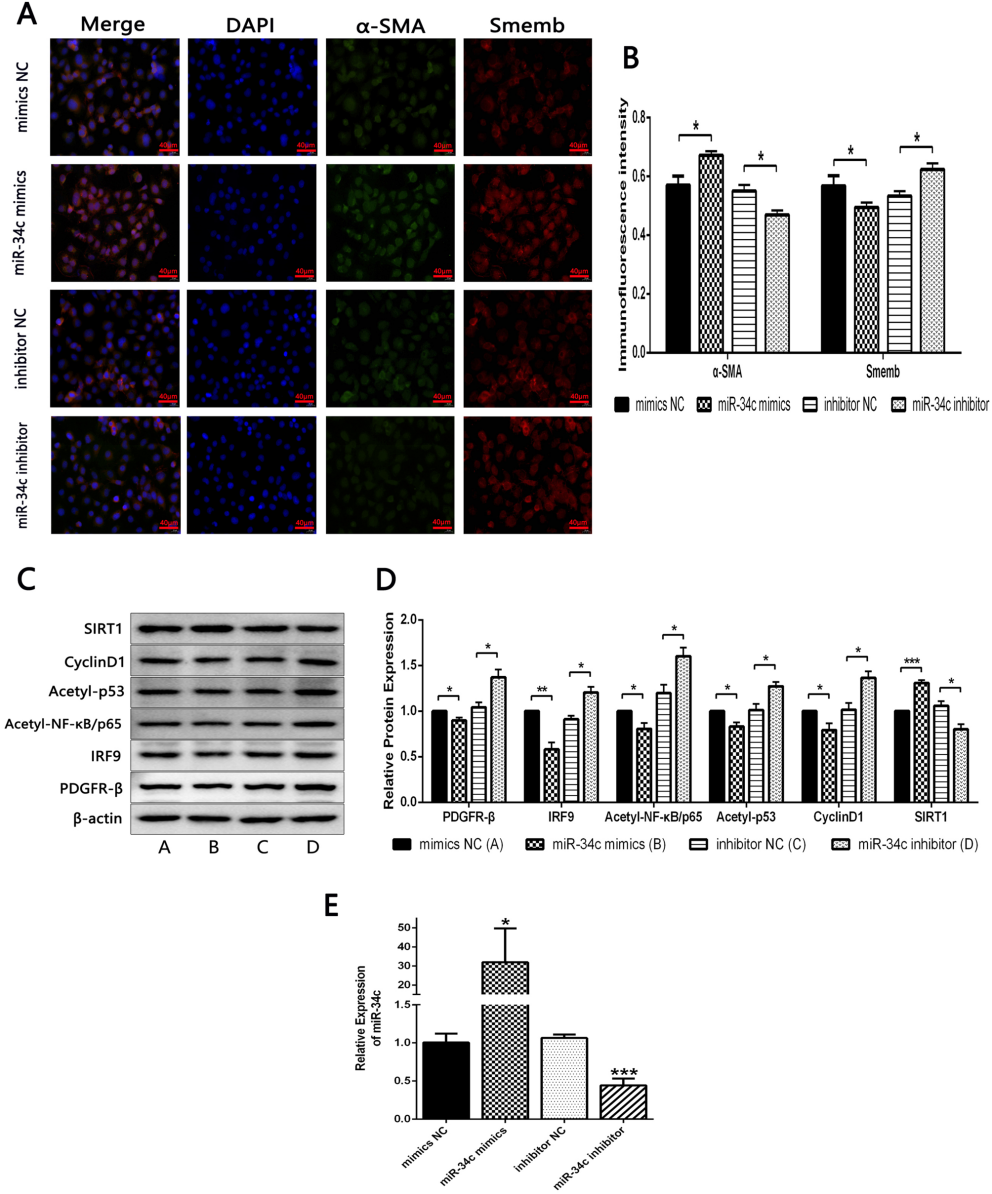
**A**, **B** Immunofluorescence of α-SMA and Smemb protein was detected in PDGF-BB-induced HA-VSMCs with overexpression or inhibition of miR-34c treatment by immunofluorescent staining. *P < 0.05 between the comparison. **C**, **D** Relative protein expressions of PDGFR-β/SIRT1 pathway were detected in PDGF-BB-induced HA-VSMCs with overexpression or inhibition of miR-34c treatment by Western blot. *P < 0.05, **P < 0.01 and ***P < 0.001 between the comparison. **E** Relative mRNA expressions of miR-34c were detected in PDGF-BB-induced HA-VSMCs with overexpression or inhibition of miR-34c treatment by RT-qPCR. *P < 0.05 and ***P < 0.001 between the comparison

To investigate whether miR-34c inhibited the phenotypic transformation of cells in PDGF-BB-induced HA-VSMCs, immunofluorescence stainings of α-SMA and Smemb were used. Results showed that the expression levels of α-SMA and Smemb were respectively significantly increased and decreased in the miR-34c mimics group, compared with the mimics NC group. Moreover, the expression levels of α-SMA and Smemb were respectively significantly decreased and increased in the miR-34c inhibitor group, compared with the inhibitor NC group (Fig. 4A, B).

Western blot results indicated that, relative to mimics NC group, the expression levels of PDGFR-β, IRF9, Acetyl-NF-κB/p65, Acetyl-p53 and CyclinD1 all had a significant downward trend, and the expression level of SIRT1 had a significant upward trend, in miR-34C mimics group. As we expected, relative to inhibitor NC group, the expression levels of PDGFR-β, IRF9, Acetyl-NF-κB/p65, Acetyl-p53 and CyclinD1 increased significantly, and the expression level of SIRT1 decreased significantly, in miR-34c inhibitor group (Fig. 4C, D).

RT-qPCR was used to detect the expression of miR-34c in overexpressed or inhibited miR-34c cells in each group. It was found that miR-34c mimics group has a significantly high expression level of miR-34c compared to mimics NC group, while miR-34c inhibitor group achieved the opposite result (Fig. 4E). In general, upregulation of miR-34c could promote the expression of α-SMA, inhibit the expression of Smemb, and inhibit the PDGFR-β/SIRT1 signaling pathway. Down-regulation of miR-34c had the opposite effect.

### PDGFR-β was the target gene of miR-34c

**Fig. 5:**
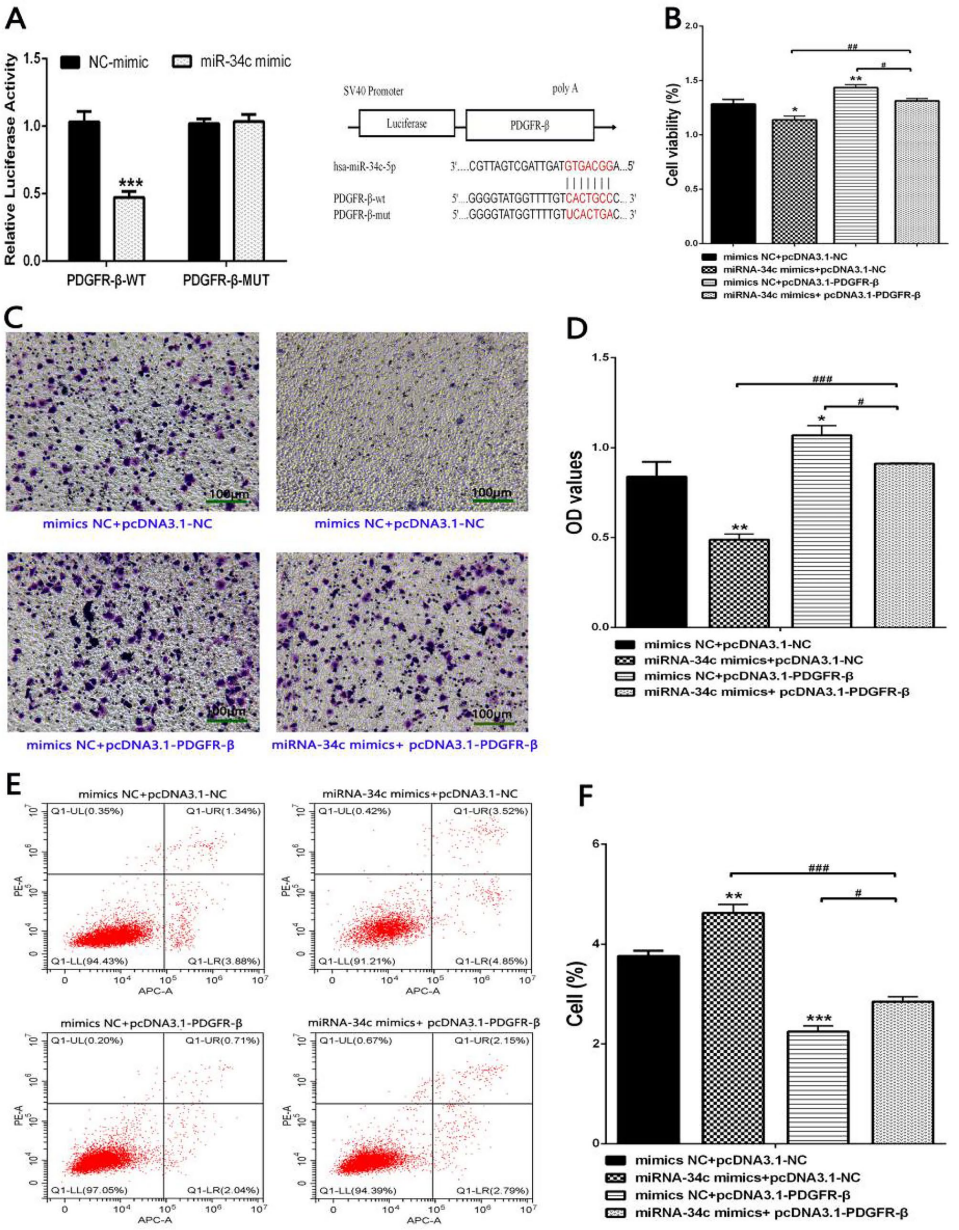

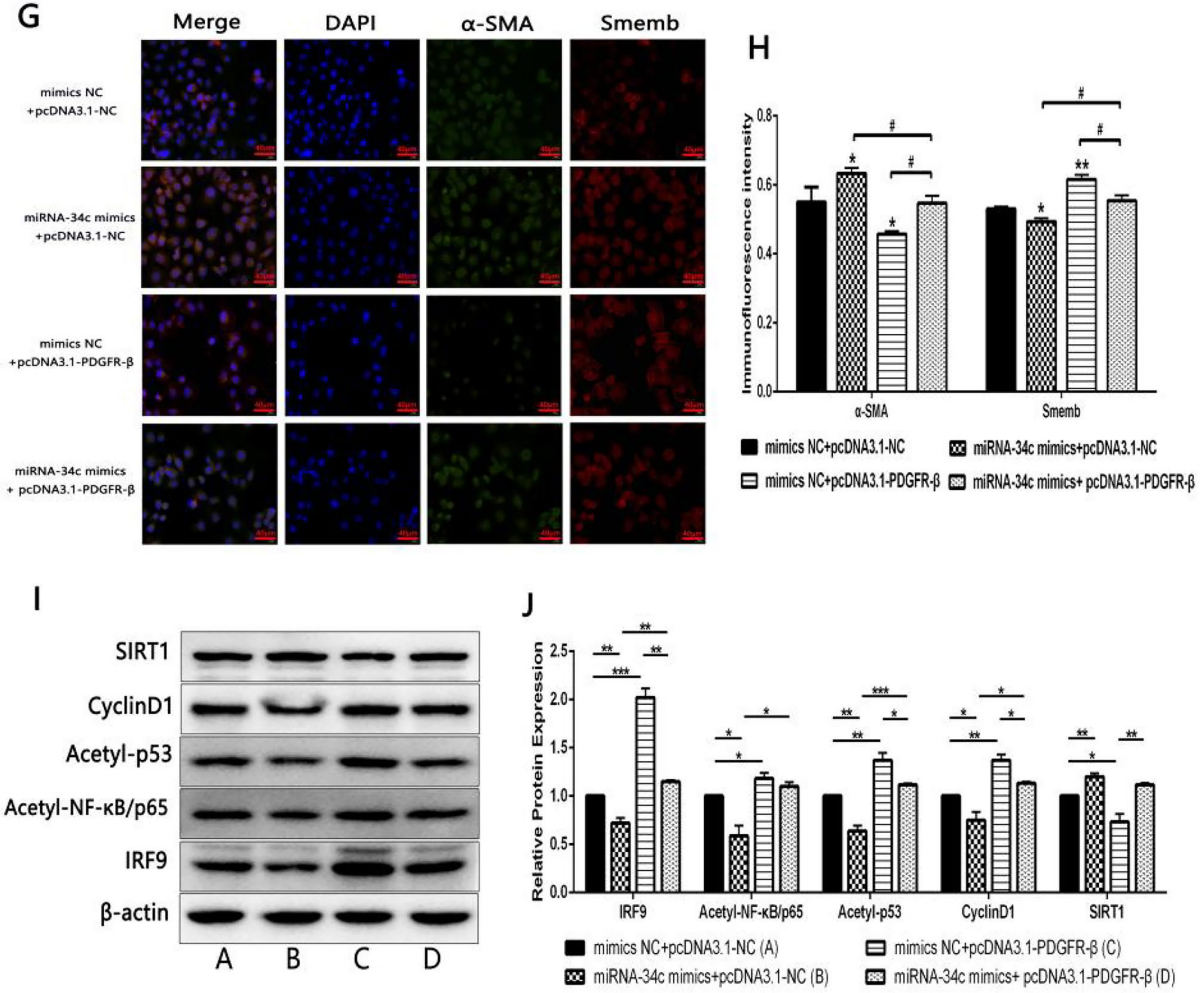
**A** Luciferase reporting assay detected that PDGFR-β 3′UTR is direct targets of miR-34c. ***P < 0.001 vs. NC-mimic group. **B** miR-34c regulating PDGFR-β inhibits PDGF-BB-induced HA-VSMCs proliferation by CCK8 assay. *P < 0.05, **P < 0.01, vs. mimics NC+pcDNA3.1-NC group; ^#^P < 0.05 and ^##^P < 0.01 between the comparison. **C**, **D** miR-34c regulating PDGFR-β inhibits PDGF-BB-induced HA-VSMCs migration by transwell chamber. *P < 0.05, ** P < 0.01, vs. mimics NC+pcDNA3.1-NC group; ^#^P < 0.05 and ^###^P < 0.001 between the comparison. **E**, **F** miR-34c regulating PDGFR-β promotes PDGF-BB-induced HA-VSMCs apoptosis by flow cytometry. **P < 0.01, ***P < 0.001, vs. mimics NC+pcDNA3.1-NC group; ^#^P < 0.05 and ^###^P < 0.001 between the comparison. **G**, **H** Immunofluorescence of α-SMA and Smemb protein was detected in PDGF-BB-induced HA-VSMCs in each group by immunofluorescent staining. *P < 0.05 and **P < 0.01, vs. mimics NC+pcDNA3.1-NC group; ^#^P < 0.05 between the comparison. **I**, **J** Relative protein expressions of PDGFR-β/SIRT1 pathway were detected in PDGF-BB-induced HA-VSMCs in each group by Western blot. *P < 0.05, **P < 0.01 and ***P < 0.001 between the comparison

To identify miR-34c targets, a bioinformatics search (Targetscan, Pictar) was performed for putative mRNA targets before experiments. 3′ UTR of human PDGFR-β contained regions (PDGFR-β nucleotides 1535-1541) that matched the seed sequences of hsa-miR-34c. Besides, in order to eliminate the combination of PDGFR-β 3′UTR and miR-34c, we use the site directed mutagenic technology to mutate the 7 bp nucleotides of PDGFR-β that are matched with the seed sequence of the miR-34c to produce the mutant type PDGFR-β 3′ mut UTR. Luciferase reporting experiment was performed to verify PDGFR-β was the direct target of miR-34c. Here, full length of wild PDGFR-β 3′UTR or PDGFR-β mut 3′UTR was inserted into luciferase vector and co-transfected with miR-34c mimic (50 mnol/L). As shown in figure, in comparison with miR-34c mimics + pmiR-NC group, there was a significant decrease of luciferase activity in the miR-34c mimics + pMIR-PDGFR-β 3′UTR group, while with no significant difference in miR-34c mimics + pMIR-PDGFR-β Mut 3′UTR group, indicating a direct interaction between the miR-34c and PDGFR-β 3′UTR, which revealed that PDGFR-β is a virtual target of miR-34c with the binding site in PDGFR-β 3′UTR (Fig. 5A). These data indicated that PDGFR-β was the target gene of miR-34c.

### miR-34c upregulation inhibited the proliferation and migration and promoted the apoptosis of PDGFR-β-induced HA-VSMCs

In order to confirm the protective effects of miR-34c regulating PDGFR-β on cell biological behaviour in PDGF-BB-induced HA-VSMCs, we investigated if miR-34c could influence proliferation, migration and apoptosis of PDGF-BB-induced HA-VSMCs. Here, PDGF-BB-induced HA-VSMCs were co-transfected with miR-34c mimics/mimics NC and pcDNA3.1-PDGFR-β/pcDNA3.1-NC to observe cell biological behaviour. Consistent with our hypothesis, a significant decrease of the proliferation and migration capabilities of PDGF-BB-induced HA-VSMCs after miR-34c overexpression or a significant increase of the proliferation and migration capabilities of PDGF-BB-induced HA-VSMCs after PDGFR-β overexpression was observed (Fig. 5B–D). Among them, we could clearly find that, compared with mimics NC+pcDNA3.1-NC group, cell proliferation and invasion capacity of miR-34c mimics+pcDNA3.1-NC group was significantly reduced, while that of mimics NC+pcDNA3.1-PDGFR-β group was significantly increased. In addition, compared with miR-34c mimics+pcDNA3.1-PDGFR-β group, we found that miR-34c mimics+pcDNA3.1-NC group and mimics NC+pcDNA3.1-PDGFR-β group respectively showed a significant decrease trend and a significant increase trend (Fig. 5B–D). Furthermore, we found that the apoptosis trend was expectedly opposite to the trend of cell proliferation and migration, where a significant increase of the apoptosis capabilities of PDGF-BB-induced HA-VSMCs after miR-34c overexpression or a significant decrease of the apoptosis capabilities of PDGF-BB-induced HA-VSMCs after PDGFR-β overexpression was observed (Fig. 5E). These data indicated that miR-34c upregulation inhibited the proliferation and migration and promoted the apoptosis of PDGFR-β-induced HA-VSMCs.

### miR-34c inhibited the phenotypic transformation of PDGF-BB-induced HA-VSMCs via inhibiting PDGFR-β/SIRT1 pathway

In order to investigate the effects of PDGFR-β regulated by miR-34c on phenotypic transformation of PDGF-BB-induced HA-VSMCs, PDGF-BB-induced HA-VSMCs were co-transfected with miR-34c mimics/mimics NC and pcDNA3.1-PDGFR-β/pcDNA3.1-NC. Immunofluorescence staining experiment indicated that the expression of α-SMA was significantly higher in miR-34c mimics+pcDNA3.1-NC group and significantly lower in mimics NC+pcDNA3.1-PDGFR-β group than in mimics NC+pcDNA3.1-NC group, whereas Smemb expression showed an opposite level. Moreover, compared to miR-34c mimics+pcDNA3.1-NC group and mimics NC+pcDNA3.1- PDGFR-β group respectively, miR-34c mimics+ pcDNA3.1-PDGFR-β group was shown a significantly lower level and a significantly higher level of SMA expression, as well as a significantly higher level and a significantly lower level of Smemb expression (Fig. 5G, H).

Western blot was used to further validate the effect of miR-34c on relative protein expressions of PDGFR-β/SIRT1 pathway in PDGF-BB-induced HA-VSMCs. The protein expression results showed that, compared with mimics NC+pcDNA3.1-NC group, the expression levels of IRF9, Acetyl-NF-κB/p65, Acetyl-p53 and CyclinD1 in miR-34c mimics+pcDNA3.1-NC group decreased significantly, while those in mimics NC+pcDNA3.1-PDGFR-β group increased significantly, with an opposite trend of SIRT1 expression in the corresponding group. In addition, the expression levels of IRF9, Acetyl-NF-κB/p65, Acetyl-p53 and CyclinD1 in miR-34c mimics+ pcDNA3.1-PDGFR-β group were significantly increased and decreased, respectively, where excluding the significance of Acetyl-NF-κB/p65 expression, when compared with miR-34c mimics+pcDNA3.1-NC group and mimics NC+pcDNA3.1-PDGFR-β group, with an opposite trend of SIRT1 expression in the corresponding group (Fig. 5I, J). These data indicated that miR-34c inhibited the phenotypic transformation of PDGF-BB-induced HA-VSMCs via inhibiting PDGFR-β/SIRT1 Pathway.

## Discussion

The transformation of smooth muscle cells from contractile to synplastic is an important marker of the progression of atherosclerotic lesions [18]. In the whole process of atherosclerosis, VSMCs are mainly involved in the middle and late stages of the disease, and migration, mass proliferation and secretion of extracellular matrix of VSMCs become important parts of the process of atherosclerosis [19, 20]. Current studies have found that phenotypic transformation of vascular smooth muscle cells, that is, the transformation of vascular smooth muscle cells from contractility to synthosis, is an indispensable initiator of abnormal migration, proliferation and excessive secretion of extracellular matrix [21]. Therefore, the regulation of phenotypic transformation of vascular smooth muscle cells is expected to be a target to inhibit or reverse the progression of atherosclerosis and even all vascular remodeling diseases and pathological conditions such as restenosis after stent implantation.

MicroRNAs, as small noncoding RNAs, are highly expressed in VSMC and are widely involved in regulating differentiation, proliferation and migration of VSMC [7, 22, 23]. A large number of studies have found that miRNAs play an important role in various biological processes such as development, organ formation, cell proliferation and apoptosis, fat metabolism, and tumor metastasis [24, 25]. Platelet derived growth factor-BB (PDGF-BB) is an important mitogenic factor locally secreted by platelet aggregation after vascular injury, which can induce the transformation of VSMCs from contractility to synplasia through interaction with different transcriptional regulators [2]. In the previous work, we found that PDGFR-β could promote phenotypic transformation of VSMCs, and the PDGFR/IRF9/SIRT1 axis was an important signaling pathway mediating VSMC phenotypic transformation [16]. Thus, we hypothesized that this axis also plays a role in PDGF-BB-induced HA-VSMCs.

Under normal physiological environment, VSMCs does not have the ability of proliferation and migration. However, after vascular endothelial injury, VSMCs can rapidly acquire the ability of proliferation, migration and ECM secretion under the stimulation of a variety of signaling molecules [26]. Early endothelial apoptosis promotes the proliferation of anti-apoptotic endothelial progenitor cells and eventually leads to clump-like lesions [27]. In the vascular media, apoptosis of HA-VSMCs cells is inhibited, which can lead to excessive cell proliferation. Previous studies have proved that PDGF-BB is the most effective and powerful proliferative molecule of VSMCs, and it is also an important pathological basis and key factor leading to atherosclerosis and restenosis [28, 29]. Consistently, in this study, we found that after induction of PDGF-BB, HA-VSMCs proliferation and migration were occurred, apoptosis was inhibited, phenotypic transformation was initiated, PDGFR-β/IRF9/SIRT1 axis was regulated, and the expressions of miR-34a, miR-34b and miR-34c were down-regulated. At present, the specific mechanism of PDGF-BB in the proliferation and migration of VSMCs has not been elucidated. Therefore, in order to understand the regulatory mechanism of VSMCs proliferation and migration, and to search for effective inhibitors of the PDGF-BB signaling pathway to reduce its proliferation-promoting effect, we designed experiments to overexpress or inhibit miR-34c, and then observed the biological behavior and phenotypic transformation of cells. Studies have shown that the differentiation between systolic and synthetic-secretory vascular smooth muscle cells mainly depends on their phenotypic marker proteins, including α-smooth muscle actin (α-SMA) and smooth muscle myosin heavy chain (Smemb), in which α-SMA is mainly expressed in the systolic cells, while Smemb is mainly expressed in the synthetic-secretory cells [30]. The results preliminarily proved that miR-34c was an effective regulatory element to inhibit the phenotypic transformation and proliferation of PDGF-BB-induced HA-VSMCs via PDGFR-β/SIRT1.

Some transcriptional factors are proved to be the key components and modulations of VSMC differentiation. Studies have found that miRNAs play an important role in the differentiation and proliferation of VSMC by regulating the expression of these transcription factors. In addition, some miRNAs are directly involved in phenotypic transformation and thickening of the VSMC [31, 32]. How to control and reverse the phenotypic transformation of vascular smooth muscle is the key to control the proliferation of smooth muscle fundamentally. To further demonstrate the targeting sites of miR-34c and PDGFR-β/SIRT1 axis, luciferase assay was used to demonstrate that PDGFR-β is direct target of miR-34c. These results were further verified by co-transfection of miR-34c mimics and PDGFR-β plasmid.

Studies have shown that SIRT1 is a histone deacetylase that regulates cell proliferation and inhibits inflammatory responses [33]. Notably, SIRT1 improves VSMC functions in atherosclerosis [34], and SIRT1 can deacetylate P53 and inhibit its expression activity [35–37]. As a tumor suppressor, P53 can inhibit cell cycle progression, differentiation and accelerate DNA repair [38, 39]. In this study, miR-34c down-regulated the Acetyl-p53 and resumed the expression of SIRT1 by regulating PDGFR-β, suggesting that miR-34c can promote SIRT1 expression, increase the activity of SIRT1, and deacetylate P53, thereby protecting PDGF-BB-induced HA-VSMCs. Moreover, in physiological and pathological conditions, the active groups of the inflammatory marker NF-κB, the p65 subunits, require multiple post-translational modifications to play the role of nuclear transcription factors, of which acetylation is an important modification in transcriptional activity [40]. The decreased expression of Acetyl-NF-κB/p65 suggested that SIRT1 reduced VSMC injury by deacetylation of NF-κB/p65. Furthermore, our previous results have also shown that activation of PDGFR-β/IRF9 pathway inhibited SIRT1, and that SIRT1 is the key mediator in maintaining VSMCs contractile phenotype [16], with consistent results that SIRT1 has a significant downward trend through the treatment of miR-34c mimics on PDGF-BB-induced HA-VSMCs. As we expected, the expression of proliferation marker Cyclin D1 was reduced under the treatment of miR-34c mimics, which further verified that the proliferation activity of PDGF-BB-induced HA-VSMCs was weakened, suggesting that PDGFR-β/IRF9/SIRT1 axis was regulated. Taken together, miR-34c targets PDGFR-β 3’UTR, strengthening the interaction among proteins involved in the PDGFR-β/SIRT1 pathway, ensuing promoting SIRT1-regulated NF-κB/p65 and p53 deacetylation and weakening the expression of IRF9 and CyclinD1.

Above all, we concluded that PDGF-BB regulated PDGFR-β/SIRT1 signal pathway in HA-VSMC cells, which lead to excessive cell proliferation and migration, and finally exacerbates phenotype transformation. MiR-34c mimics inhibited cell proliferation, migration and phenotypic transformation, facilitated the apoptosis, and reversed the progress of PDGFR-β/SIRT1 pathway. All of these results suggested that miR-34c was a key regulatory factor protecting PDGF-BB-induced HA-VSMCs.

## Supplementary Information

Below is the link to the electronic supplementary material.

Supplementary file1 (RAR 13119 kb)

## Data Availability

The datasets used during the present study are available from the corresponding author on reasonable request.

## Abbreviations

VSMCs: Vascular smooth muscle cells
PDGF-BB: Platelet-derived growth factor-BB
HA-VSMCs: Human aortic vascular smooth muscle cells
SMC: Smooth muscle cells
α-SMA: α-Smooth muscle actin
Smemb: Smooth muscle myosin heavy chain
